# Apelin-13 Protects Neurons by Attenuating Early-Stage Postspinal Cord Injury Apoptosis In Vitro

**DOI:** 10.3390/brainsci12111515

**Published:** 2022-11-08

**Authors:** Taotao Lin, Yujie Zhao, Shengyu Guo, Zhengru Wu, Wenwen Li, Rongcan Wu, Zhenyu Wang, Wenge Liu

**Affiliations:** Department of Orthopedics, Fujian Medical University Union Hospital, Fujian Medical University, Fuzhou 350001, China

**Keywords:** apelin-13, transcriptome sequencing, spinal cord injury, autophagy, apoptosis

## Abstract

Apelin is a 77-amino-acid peptide that is an endogenous ligand for the G protein-coupled receptor APJ (Apelin receptor, APJ). Apelin-13, as the most bioactive affinity fragment of apelin, plays a role in energy metabolism, myocardial ischemia-reperfusion injury, and the regulation of the inflammatory response during oxidative stress, but its role in spinal cord injury is still unclear. This research identified and verified the differential expression of apelin in rat spinal cord injured tissues and normal spinal cord tissues by transcriptome sequencing in vivo and proved that apelin-13 protects neurons by strengthening autophagy and attenuating early-stage postspinal cord injury apoptosis in vitro. After constructing the model concerning a rat spinal cord hemisection damage, transcriptome sequencing was performed on the injured and normal spinal cord tissues of rats, which identified the differentially expressed gene apelin, with qRT-PCR detecting the representative level of apelin. The oxygen-glucose deprivation (OGD) model of PC12 cells was constructed in vitro to simulate spinal cord injury. The OGD injury times were 2 h, 4 h, 6 h, 8 h, and 12 h, and the non-OGD injury group was used as the control. The expression of apelin at each time point was observed by Western blotting. The expression of apelin was the lowest in the 6 h OGD injury group (*p* < 0.05). Therefore, the OGD injury time of 6 h was used in subsequent experiments. The noncytotoxic drug concentration of apelin-13 was determined with a Cell Counting Kit-8 (CCK-8) assay. An appropriate dose of apelin-13 (1 μM) significantly improved cell survival (*p* < 0.05). Thus, subsequent experiments selected a concentration of 1 μM apelin-13 as it significantly increased cell viability. Finally, we divided the experimental groups into four groups according to whether they received drugs (1 μM apelin-13, 24 h) or OGD (6 h): (1) control group: without apelin-13 or OGD injury; (2) apelin-13 group: with apelin-13 but no OGD injury; (3) OGD group: with OGD injury but without apelin-13; and (4) OGD + apelin-13 group: with apelin-13 and OGD injury. The TUNEL assay and flow cytometry results showed that compared with the OGD group, apoptosis in the OGD+Apelin-13 group was significantly reduced (*p* < 0.001). Determination of cell viability under different conditions by CCK-8 assay results displays that Apelin-13 can significantly improve the cell viability percentage under OGD conditions (*p* < 0.001). Western blotting results showed that apelin-13 decreased the expression ratios of apoptosis-related proteins Bax/Bcl-2 and cleaved-caspase3/caspase3 (*p* < 0.05), increasing the key to Beclin1-dependent autophagy pathway expression of the protein Beclin1. This finding indicates that apelin-13 protects neurons by strengthening autophagy and attenuating early-stage postspinal cord injury apoptosis in vitro.

## 1. Introduction

Spinal cord injury (SCI) is a serious infirmity of the nervous system which impairs sensory and motor functions, causing multiple organ dysfunction. It causes serious damage to patients’ physical functions, psychology, and social participation ability and places a heavy economic and spiritual burden on families, the medical system, and society [1]. The pathophysiological progression of SCI has two phases. The initial phase of SCI is usually resulted from mechanical injuries, such as intense compression, tearing as well as shearing. The second stage of SCI is to induce the hypoxic and ischemic extracellular environment around neurons [2]. Secondary spinal cord ischemia-hypoxia injury is an important pathological process of SCI. The occurrence and development of SCI are closely related to changes in the peripheral environment of spinal cord neurons and the apoptosis of spinal cord neurons themselves. An in-depth study of the molecular mechanism of spinal cord neuron apoptosis caused by ischemia-hypoxia injury offers fresh perspectives to the treatment of SCI [3,4]. Because of their nonrenewable nature, neurons undergo a series of pathophysiological changes after injury and eventually undergo apoptosis, which is irreversible. Therefore, inhibition of neuronal apoptosis is critical for the treatment of SCI.

Apelin is a 77-amino-acid peptide that is an endogenous ligand for the G protein-coupled receptor APJ. It can be divided into four shorter active forms, apelin-12, apelin-13, apelin-17, and apelin-36 [5] included. All proteins bind to APJ receptors, and different conformational states of the receptors may affect protein activity [6], of which apelin-13 is considered to be the most bioactive affinity fragment [7]. The apelin-APJ system exists throughout in the center of the nervous system and peripheral organs and tissues, such as the heart, kidney, liver, and adipose tissue, and performs a wide range of functions, such as energy metabolism, myocardial ischemia-reperfusion injury and regulation of the inflammatory response during oxidative stress [8,9,10,11,12,13,14]. More and more studies show that Apelin-13 acts as an essential protective part of the central nervous system [15,16,17]. Bao et al. [15] found that apelin-13 inhibits traumatic brain injury-induced brain damage by suppressing autophagy. Khaksari M et al. [16] found that apelin-13 provides protection against transient focal cerebral ischemia by reducing the volume of cerebral infarction, reducing brain edema, and inhibiting apoptosis. Yan et al. [17] found that apelin-13 inhibits apoptosis and plays a neuroprotective role in cerebral ischemia-reperfusion injury. But its role in spinal cord injury is still unclear.

In this research, we identified and verified the differential expression of apelin in rat spinal cord injured tissues and normal spinal cord tissues by transcriptome sequencing in vivo and proved that apelin-13 protects neurons by strengthening autophagy and attenuating early-stage postspinal cord injury apoptosis in vitro.

## 2. Materials and Methods

### 2.1. Animals

Adult male Sprague-Dawley rats weighing 200 to 220 g were used. Prior to the experiment, the animals were housed in a room for at least 7 days at 12 h in temperature regulation (23–25 °C) and humidity control (50% relative humidity). Food was not allowed, but water can drink freely all night prior to surgery. According to the guidelines of the Animal Ethics Committee of Fujian Medical University (Fuzhou, China) (No: IACUC FJMU 2022-0717), we conducted all the experiments.

### 2.2. Establishment of the Spinal Cord Injury Model

As mentioned in the research, the SCI model (the T9 lateral hemisection surgical procedure) was induced following the method described by Wu et al. [18].

In simple terms, before the middle line of the back skin was incised, the anesthesia, hair removal, and disinfection with 70% alcohol had been performed.

The subcutaneous tissue which ranged from T8 to T10 was cut directly open, as well as the muscles and tissue covering the spine to reveal the T9 lamina. The spinal cord on the right side of the T9 was then hemidissected using an ophthalmic iris knife. The muscle layer was stitched and the skin was again. The sham group only exposed the spinal cord by laminectomy following the above procedure and did not manage the spinal cord. To ensure consistency of the injury, all procedures were performed by the same experienced surgeon.

### 2.3. RNA Extraction, Library Construction, and Sequencing

To mimic acute events, rats (*n* = 3 per group) were anesthetized and sacrificed 6 h after SCI and immediately after sham surgery for further RNA sequence analysis.

Transcriptomic analysis was performed by SECCO Health Technology Co., Ltd. (Wuhan, China). All the RNA from the above samples was insulated in accordance with instructions of the manufacturer employing Trizol kit (Promega, Madison, WI, USA). Residual Treatment with RNase-free DNase I (Takara Bio, Kusatsu, Japan) then eliminated DNA at 37 °C up to 30 min. A 2100 Bioanalyzer (Agilent Technologies, Santa Clara, CA, USA) as well as the RNase-free agarose gel electrophoresis were applied to verify the quality of RNA and detect the RNA, respectively. Next, the oligo-dt beads (Qiagen) were adopted to insulate the Poly (A) mRNA. By adding a fragment buffer, all mRNA was decomposed into short fragments. Reverse transcription using random hexamer primers to generate first-strand cDNA, and second-strand DNA was synthesized adopting RNase H and DNA polymerase i. The QIAquick PCR extraction kit was chosen to cleanse the cDNA chips. It was then cleansed with EB buffer, end-repaired poly (A) was added, and connected to a sequence adapter. The final cDNA library was constructed by agarose gel electrophoresis and extraction of cDNA from the gel. The enriched cDNA fragment was purified by PCR. The cDNA library was sequenced on the Ili Mumina Sequencing platform (Illumination aHiSeq™2000) using Gene Denovo (Guangzhou, China) peer technology.

### 2.4. Differentially Expressed Genes (DEGs) and Function Enrichment Analyses

After calculating every gene’s expression level, differential expression analysis was performed using edgeR [19]. The threshold of the *p*-value in multiple tests was determined by adopting the false discovery rate (FDR), and a threshold of

FDR ≤ 0.01 and an absolute value of log2Ratio ≥ 1 were used to estimate the consequence of gene expression differences.

GO and KEGG enrichment analysis of differentially expressed genes was conducted by adopting the method depicted by Zhang et al. [20]. GO terms as well as KEGG pathways were significantly enriched in deg.

### 2.5. Quantitative Real-Time Polymerase Chain Reaction (qRT-PCR) Analysis

**Figure 1:**
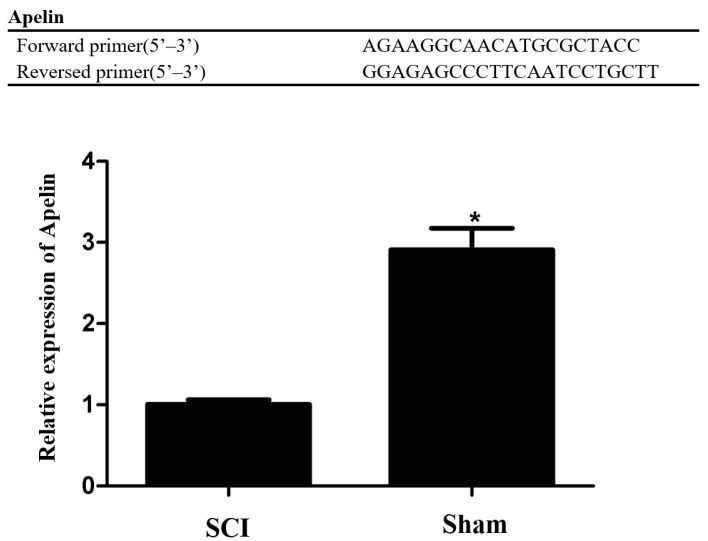
Primer sequences of Apelin and qRT-PCR results showed that the expression of Apelin in spinal cord injury tissues was down-regulated compared with normal rat spinal cord tissues. * *p* < 0.05.

The qRT-PCR method was adopted to verify the accuracy of RNA-seq data. Real-time PCR analysis of target RNA and internal parameters in every sample was performed separately using an ABI Prism^®^7500 Sequence Detection System (Applied BioSystems, Foster, CA, USA). All the RNA was reverse transcribed, and first-strand cDNA was synthesized adopting Pinben RT kit synthesis (perfect real-time; TaKaRa, Osaka, Japan). The 2^−ΔΔCT^ approach was adopted to analyze the data. The qRT-PCR amplification method was indicated as below: detection at 95 °C for 10 min, with 40 PCR cycles following, detection at 95 °C for 10 s, as well as detection at 60 °C for 60 s (collected by fluorescence emission). After completing the amplification reaction, the following denaturation process is performed: 95 °C for 10 s, 60 °C for 60 s, and 95 °C for 15 s. Melting curves of PCR products were established by slowly increasing the temperature from 60 °C to 99 °C at a rate of 0.05 °C/s. The PCR primers used to amplify Apelin are shown in Figure 1.

### 2.6. Cell Culture, Differentiation, and Oxygen-Glucose Deprivation (OGD) Model and Treatment

PC12 cells have formerly been adopted for in vitro research of SCI [21,22], which are derived from the rat adrenal pheochromocytoma cell line and resemble a neuron. They were purchased from the Chinese Academy of Sciences Cell Bank (Shanghai, China) and cultured at Roswell Park Memorial Institute 1640 Medium (Gibco, Petaluma, CA, USA) containing 10% horse serum (Gibco, Petaluma, CA, USA, NZ origin), 5% fetal bovine serum (FBS; Gibco, Petaluma, CA, USA, Brazilian origin) along with 1% penicillin/streptomycin (Gibco, Petaluma, CA, USA), 37 °C, carbon dioxide 5%.

PC12 cells were seeded on 1 × 10^6^ cells/well poly-l-lysine-coated 6 cm plates as well as cultured for 24 h. RPMI-1640 containing 10% FBS, 50 ng/mL NGF (Sangon Biotech, Shanghai, China), and 1% penicillin/streptomycin was used to replace the culture medium. Once every two days until day 5, shifting the medium when the neurite length was longer than that of a single cell body.

OGD model induces NGF-differentiated PC12 cell death and apoptosis, mimicking ischemic spinal cord injury in vivo [23]. PBS was adopted to wash the cells, then cultured using a Form Series II CO dioxide incubator (Thermo, Allentown, PA, USA), 1% O_2_, 5% CO_2,_ and 95% N_2_ in serum-free Hanks’ equilibrated salt solution (Gibco, Petaluma, CA, USA) for 6 h to stimulate early cell damage.

### 2.7. Cell Viability Assay

With different concentrations of apelin-13 added, PC12 cell viability was determined by the CCK-8 method. PC12 cells were briefly seeded in a 96-well plate (1.5 × 10^4^ per well), with 100 μL of complete medium added, and cultured for 24 h. Then, 10 μL of CCK-8 solution (Michito Lab, Kyushu, Japan) was added to each well plate, as well as incubated for 2 h. Eventually, absorbance at 450 and 630 nm adopting a microplate reader (BioTek, Winooski, VT, USA).

### 2.8. Western Blot Analysis

Total protein was extracted from cells and tissues, as well as protein concentration was determined using a BCA detection kit (Boster Biotech, Wuhan, China). Proteins were separated by electrophoresis on a sulfuric acid dodd sodium-polyacrylamide gel and transferred to a polyvinylidene fluoride membrane. Five percent thin milk powder blocked the membranes for 2 h at room temperature, with antibodies incubating to Apelin (1:1000, Abcam, Cambridgeshire, England), Beclin 1 (1:1000, Cell Signaling Technology, Danvers, MA, USA), Bax (1:1000, Cell Signaling Technology, Danvers, MA, USA), Bcl-2 (1:1000 Abcam, Cambridgeshire, England), cleaved-caspase3 (1:500 Abcam, Cambridgeshire, England), caspase3 (1:5000 Abcam, Cambridgeshire, England) and β-actin (as a gel-loading control, 1:1000, Cell Signaling Technology, Danvers, MA, USA) was incubated at 4 °C for 12 h. Membranes were incubated with HRP-conjugated secondary antibody (1:5000, Dingguo, Beijing, China) at room temperature for 2 h, and then immunolabeled bands were visualized using enhanced chemiluminescence reagent (Beyotime Biotech, Hangzhou, China). Protein expression levels were detected by ImageJ software (NIH, Bethesda, MD, USA).

### 2.9. TdT-Mediated dTUP Nick End Labeling (TUNEL) Staining

TUNEL reaction mixture (Roche, Basel, Switzerland) was used to fix, block and incubate with PC12 cells for 1 h at 37 °C, with DAPI counterstaining the nuclei. The proportion of TUNEL-positive cells in each group was counted using a fluorescence microscope (Leica, Heidelberg, Germany).

### 2.10. Flow Cytometry

After the treatment, centrifugation at 1200 rpm for 3 min was performed to harvest the cells, with PBS washing twice. The harvested cells were resuspended in FITC-labeled Annexin V (5 μL; BD Bioscience, San Diego, CA, USA) as well as PI (5 μL; BD bioscience, CA, USA) in darkness for 5 min, with PBS washing three times. Finally, flow cytometry (FACSCalibur; BD bioscience, CA, USA) was adopted to calculate the cell apoptosis rate.

### 2.11. Statistical Analysis

We analyzed all data expressed as the mean ± standard deviation of at least three independent experiments using SPSS software (version 25.0, Chicago, IL, USA) and Prism v8.0 software (GraphPad, San Diego, CA, USA), unless otherwise stated in the figure legends. For three independent experiments, One-way ANOVA and Tukey’s multiple comparisons test were used to compute p-values. Data are displayed as mean ± SEM. Statistical significance is as follows: **** *p* < 0.0001, *** *p* < 0.001, ** *p* < 0.01, *p < 0.05.

## 3. Results

### 3.1. Identification of Differentially Expressed mRNAs in the Rat Spinal Cord Following SCI

**Figure 2:**
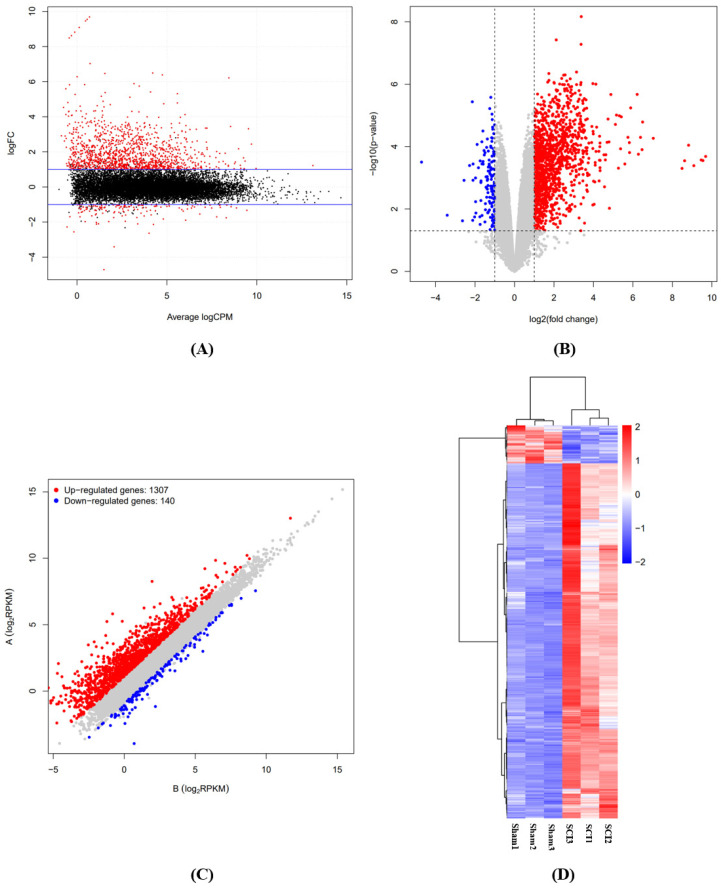
(**A**) MA plot of mRNA expression between the sham group and SCI group. The red dots show >two-fold variation in the expression of mRNAs between the two groups (*n* = 3 per group). (**B**) Volcano plot of mRNAs expression between the sham group and SCI group. The red dots indicate up-regulated mRNAs. The blue dots indicate down-regulated mRNAs. (**C**) Scatter plot of mRNA expression between the sham group and SCI group. The red dots indicate up-regulated mRNAs. The blue dots indicate down-regulated mRNAs. (**D**) Hierarchical clustering analysis of mRNAs that were prominently differentially expressed (*p*-value ≤ 0.05 and fold-change ≥ 2) between the sham group and SCI group.

RNA-seq was adopted to study the expression profile of mRNA in the spinal cord after SCI. We identified the differentially expressed mRNAs between the spinal cord injury rat tissue and the spinal cord tissue of normal rats through an MA plot (Figure 2A), volcano plot (Figure 2B), scatter plot (Figure 2C), and hierarchical clustering, indicating that the mRNA expression levels were distinguishing (Figure 2D). Compared with the sham-operated group, there were 1447 significantly differentially expressed mRNAs in the spinal cord after SCI (FC ≥ 2 and *p*-value ≤ 0.05), of which 1307 were up-regulated and 140 were down-regulated.

### 3.2. Analyses of GO and KEGG Pathways in the Differentially Expressed mRNAs

**Figure 3:**
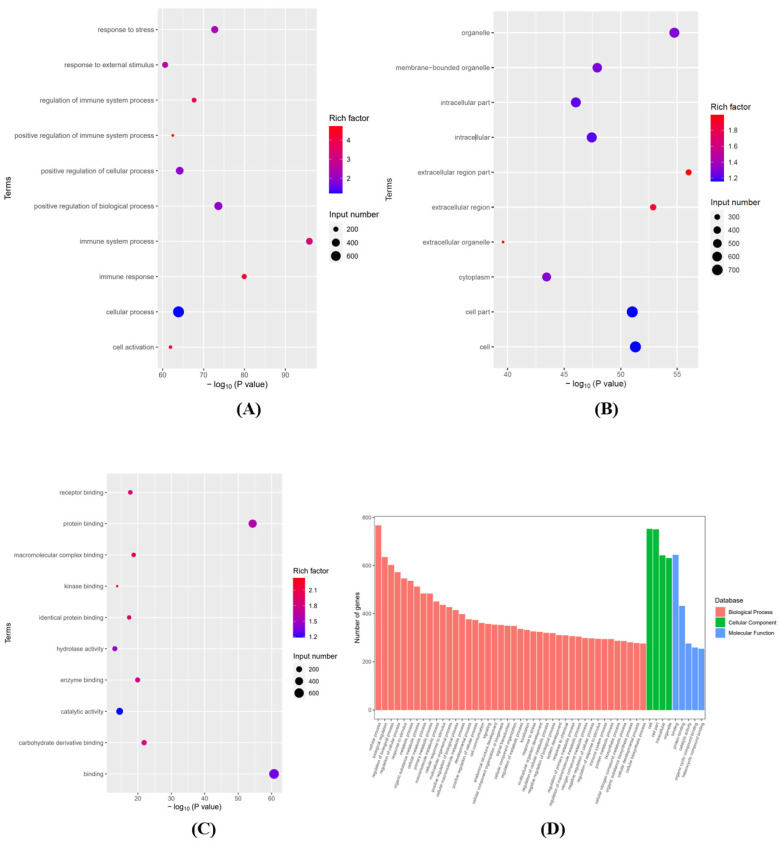
(**A**) The top 10 significantly enriched BP terms of up-regulated mRNA. (**B**) The top 10 significantly enriched CC terms of up-regulated mRNA. (**C**) The top 10 significantly enriched MF terms of up-regulated mRNA. (**D**) Integrated some significant BP, CC, and MF terms and shows them in a bar graph.

**Figure 4:**
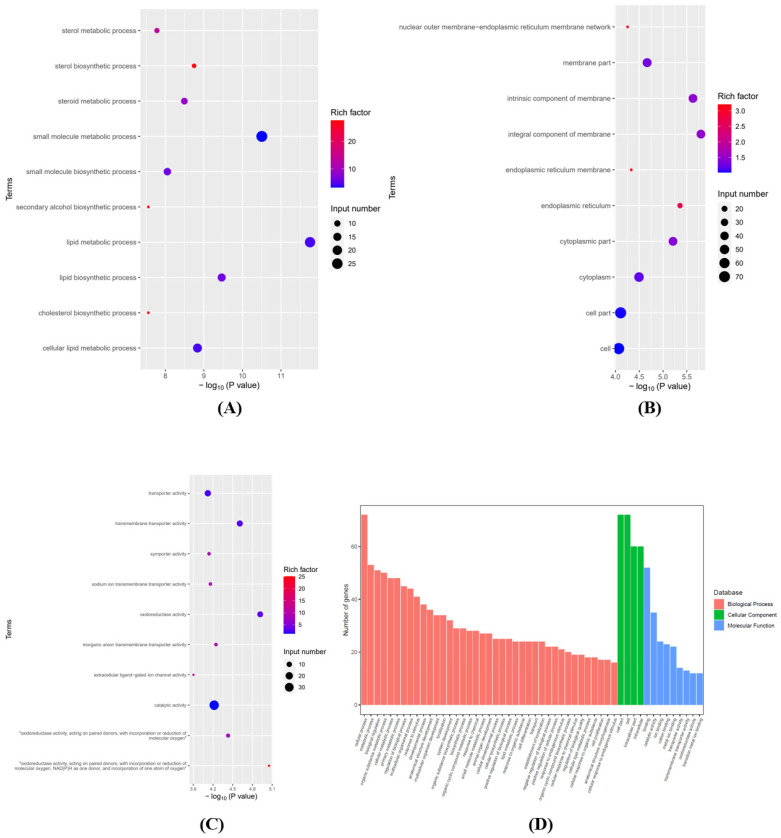
(**A**) The top 10 significantly enriched BP terms of down-regulated mRNA. (**B**) The top 10 significantly enriched CC terms of down-regulated mRNA. (**C**) The top 10 significantly enriched MF terms of down-regulated mRNA. (**D**) Integrated some significant BP, CC, and MF terms and shows them in a bar graph.

To investigate the potential functions of mRNAs after SCI, we analyzed the GO and KEGG pathways based on differentially expressed mRNAs. Graphene oxide analysis mainly includes three main areas: “Biological Process” (BP), “Cellular Composition” (CC), and “Molecular Function” (MF). The top 10 enriched GO entries for up- and down-regulation were sorted and chosen by enrichment score (−log10(*p*-value)). Figure 3A–C lists the top 10 items that were significantly enriched for the up-regulation of BP, CC, and MF. Figure 3D integrates some important BP, CC, and MF terms and displays them in a histogram. Figure 4A–C lists the top 10 terms for BP, CC, and MF that were significantly enriched in downregulated mRNAs. Figure 4D integrates some important BP, CC, and MF terms and displays them in a histogram.

**Figure 5:**
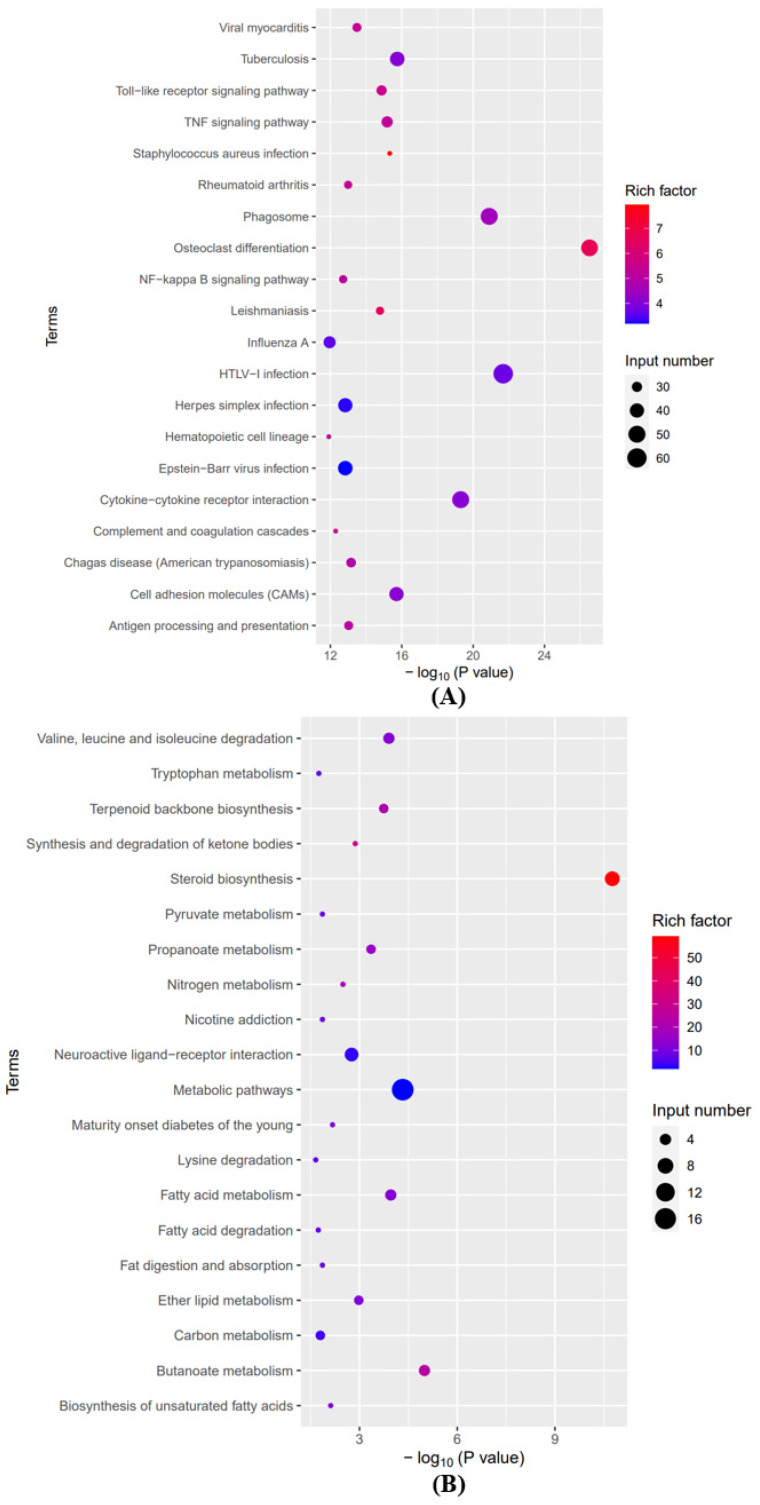
(**A**) The top 20 pathways associated with the up-regulated mRNAs through KEGG pathway analysis. (**B**) The top 20 pathways associated with the down-regulated mRNAs through KEGG pathway analysis.

The results of the KEGG pathway analysis displayed that the pathways involved in the physiological and pathological processes of spinal cord injury were significantly enriched. Through KEGG analysis, 132 pathways related to up-regulated mRNA function were identified. The top 20 pathways associated with these mRNAs are shown in Figure 5A. Through KEGG analysis, 26 pathways related to down-regulated mRNA function were identified. The top 20 pathways associated with these mRNAs are displayed in Figure 5B.

### 3.3. Selection and Expression Verification of the Differentially Expressed mRNAs

According to the functional description of differentially expressed mRNAs and GO and KEGG pathway analysis results, we selected apelin, a gene whose expression is downregulated in spinal cord injury tissues, for verification. qRT-PCR was adopted to verify the expression level of apelin. As shown in Figure 1, the expression of apelin in spinal cord injury tissues was downregulated compared with that in normal rat spinal cord tissues. The results of the qRT-PCR validation were compatible with those of transcriptome sequencing.

### 3.4. Effect of Different OGD Injury Times on Apelin Expression in the OGD Model of PC12 Cells In Vitro

**Figure 6:**
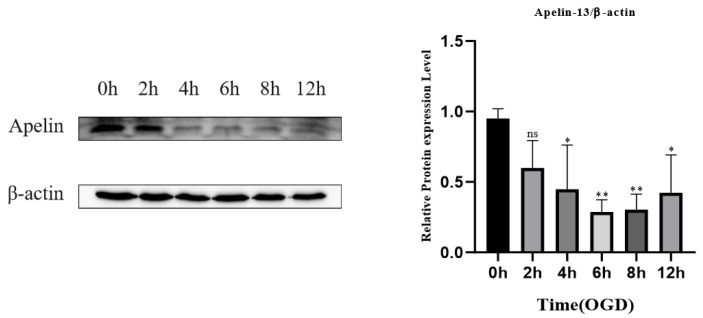
The results of the western blot showed that the expression level of Apelin decreased first and then increased, and the expression level was the lowest in the 6 h OGD injured group. For three independent experiments, One-way ANOVA and Tukey’s multiple comparisons test were adopted to compute *p*-values. ** *p* < 0.01, * *p* < 0.05. ns, not significant.

Oxygen-glucose deprivation (OGD) of neurons is regarded as an extracellular environment model after SCI in animals. OGD-induced NGF-differentiated PC12 cell death and apoptosis to simulate ischemic SCI in vivo [23]. In our study, OGD injury times of 2 h, 4 h, 6 h, 8 h, and 12 h were used, and the non-OGD injury group was used as the control. After extracting the proteins from each group, we observed the expression of apelin at each time point by Western blotting. The results showed that the expression level of apelin in the OGD injury group was significantly decreased at 4 h, 6 h, 8 h, and 12 h compared with that in the non-OGD injury group (*p* < 0.05). The expression of apelin in the 2 h OGD-injured group was slightly no higher than in the non-OGD-injured group, but the difference was not significant (*p* > 0.05). Overall, the expression level of apelin first decreased and then increased, and the expression level was the lowest in the 6 h OGD-injured group (Figure 6). Therefore, the OGD injury time of 6 h was used in subsequent experiments.

### 3.5. The Cytotoxicity of Apelin-13 at Different Concentrations Was Determined by CCK-8 Assay

**Figure 7:**
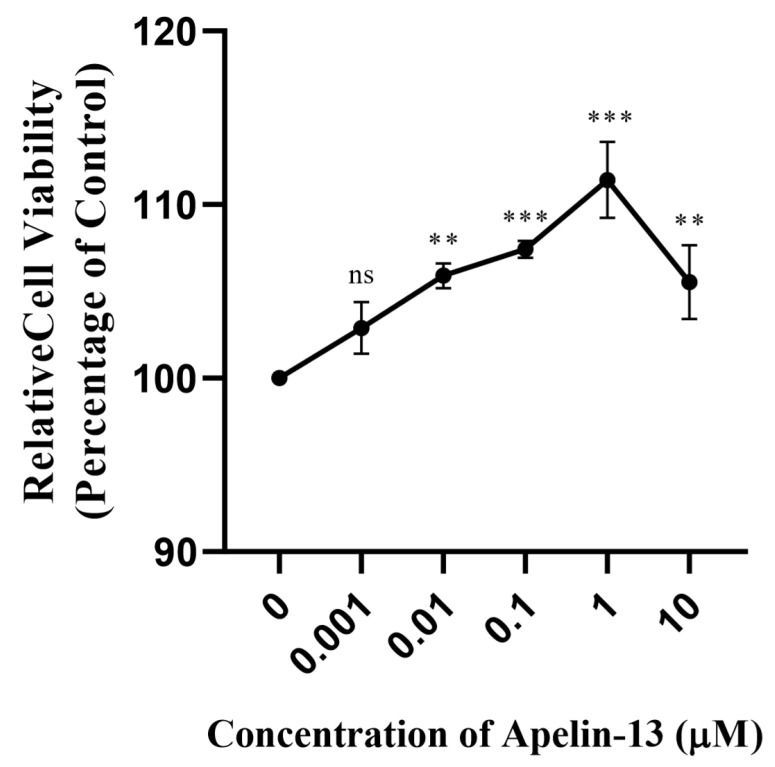
Quantitative analysis of relative cell viability with various concentrations for Apelin-13 by CCK-8’s measurement. *** *p* < 0.001, ** *p* < 0.01. ns, not significant.

The results display that when the final concentration of apelin-13 in the medium was 0.01 μM, 0.1 μM, 1 μM or 10 μM for 24 h, PC12 cells were not significantly inhibited, indicating that apelin-13 has no obvious cytotoxicity. Furthermore, the results showed that an appropriate dose of apelin-13 (1 μM) significantly increased cell viability (*p* < 0.05) (Figure 7 and Table 1). Thus, subsequent experiments selected a concentration of 1 μM apelin-13 as it significantly increased cell viability.

### 3.6. Apelin-13 Enhanced Autophagy and Decreased Apoptosis In Vitro Model

**Figure 8:**
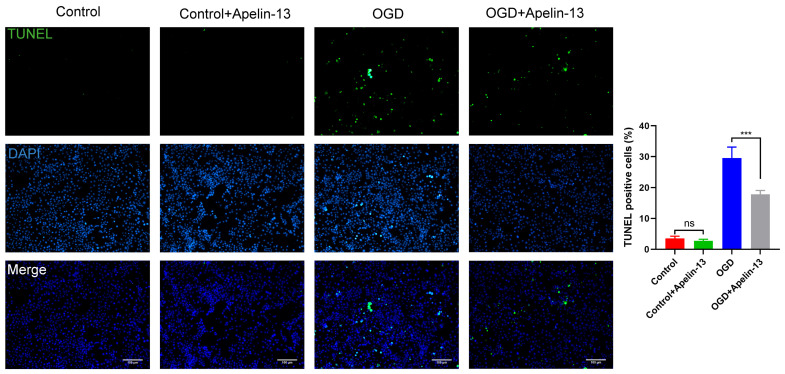
The TUNEL assay results showed that compared with the OGD group, apoptosis in the OGD+Apelin-13 group was significantly reduced. Scale bar = 100 μm. *** *p* < 0.001. ns, not significant.

**Figure 9:**
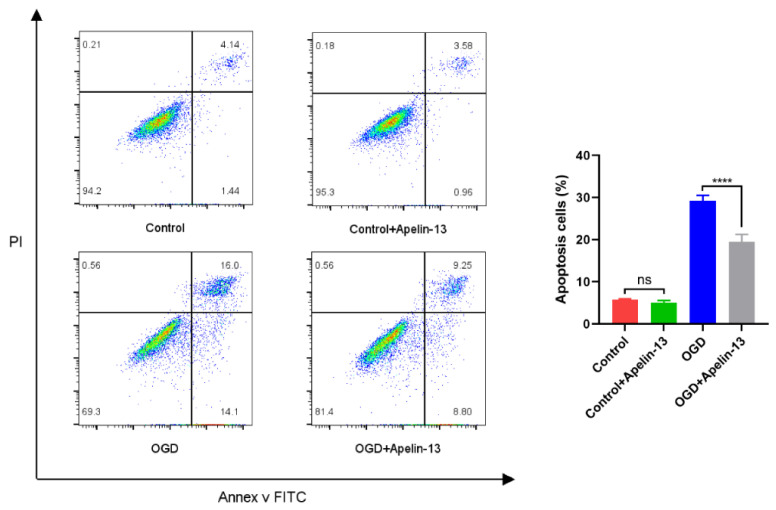
The flow cytometry results showed that compared with the OGD group, apoptosis in the OGD+Apelin-13 group was significantly reduced. **** *p* < 0.0001. ns, not significant.

**Figure 10:**
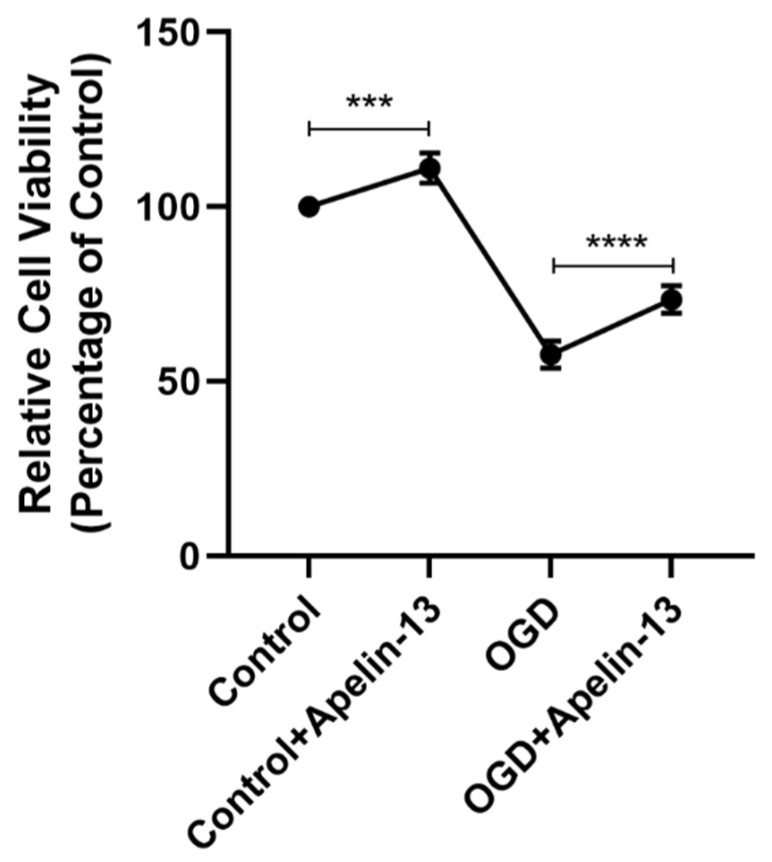
Determination of cell viability under different conditions by CCK-8 assay results showed that Apelin-13 can significantly improve the cell viability percentage under OGD conditions. **** *p* < 0.0001, *** *p* < 0.001.

**Figure 11:**
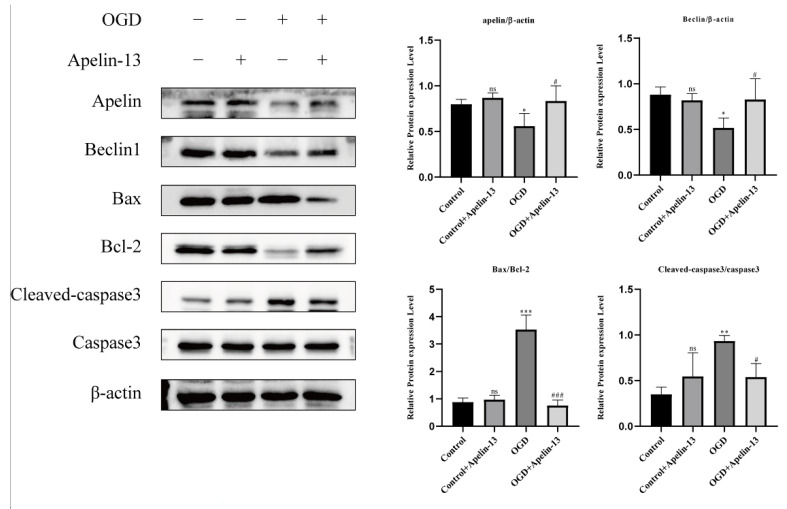
The results of the western blot in Apelin, autophagy, and apoptosis proteins in different groups. For three independent experiments, One-way ANOVA and Tukey’s multiple comparisons test were used to calculate *p*-values. * Represents that compared with control group and *** *p* < 0.001, ** *p* < 0.01, * *p* < 0.05. # Represents that compared with OGD group and # *p* < 0.05, ### *p* < 0.001. ns, not significant.

In our study, we divided the research groups into four groups according to whether they received drugs (1 μM apelin-13, 24 h) or received OGD (6 h): (1) control group: without apelin-13 or OGD injury; (2) apelin-13 group: with apelin-13 but no OGD injury; (3) OGD group: with OGD injury but without apelin-13; (4) OGD+apelin-13 group: with apelin-13 and OGD injury. The TUNEL assay and flow cytometry results showed that compared with the OGD group, apoptosis in the OGD+Apelin-13 group was significantly reduced (*p* < 0.001) (Figure 8 and Figure 9). Determination of cell viability under different conditions by CCK-8 assay results displays that Apelin-13 can significantly improve the cell viability percentage under OGD conditions (*p* < 0.001) (Figure 10 and Table 2). The results of western blot display that apelin-13 reduced the ratio of the expression of the apoptosis-related proteins Bax/Bcl-2 and cleaved-caspase3/caspase3 (*p* < 0.05) and increased the expression of Beclin1, a key protein in the Beclin1-dependent pathway of autophagy (*p* < 0.05) (Figure 11). This indicates that apelin-13 protects neurons by strengthening autophagy and attenuating early-stage postspinal cord injury apoptosis in vitro.

## 4. Discussion

Annually, there are approximately 10.4–83 cases of SCI per million people worldwide, with approximately 2.5 million people experiencing chronic SCI symptoms [24,25]. Although a series of research efforts worldwide have been focused on SCI, there is no effective breakthrough to cure SCI. Therefore, it is necessary to further explore the etiological causes and to find effective treatments. Spinal cord oxidative stress and neuronal apoptosis are the major causes of SCI phase II [1,26]. Therefore, this study evaluated the methods to cut down apoptosis. This research concentrated on apelin, which is related to apoptosis. First, after constructing a rat spinal cord hemisection injury model, transcriptome sequencing was performed on the injured and normal spinal cord tissues of rats, which identified the differentially expressed gene apelin, and the expression level of apelin was verified by qRT-PCR. Then, an oxygen-glucose deprivation (OGD) model of PC12 cells was constructed in vitro to simulate spinal cord injury, and we found that apelin-13 enhanced autophagy and cut down apoptosis in the vitro model. These findings further demonstrated that apelin-13 could be a potential treatment for spinal cord injury.

It has been noticeable that apoptosis is the fundamental cause of spinal neuronal death in human tissues and in animal models [27]. Apoptosis is mainly mediated by Bcl-2 and the caspase family. Bax/Bcl-2/cleaved-caspase3 apoptosis signaling pathway as a regulator of apoptosis and survival is involved in various diseases [28,29]. Appropriate ratios of Bax (pro-apoptotic signal) and Bcl-2 (anti-apoptotic molecule) are also significant for prolonging mitochondrial structure and function [30]. Increased Bax/Bcl-2 and cleaved-caspase3/caspase3 ratios may lead to caspase-dependent apoptosis [31]. In our study, we found that apelin-13 significantly reduced the apoptosis of PC12 cells caused by OGD injury, as shown by the TUNEL assay, flow cytometry results, Cell viability assay, and the reduced expression of the apoptosis-related proteins Bax/Bcl-2 and the cleaved-caspase3/caspase3 ratios.

Several studies suggest that autophagy protects spinal cord neurons from sci-induced early apoptosis [32,33,34]. The BECN1 gene plays a vital part in the process of autophagy, as well as its protein product Beclin1 can bind to a variety of autophagy-related proteins to form a core complex, promote the recruitment and assembly of phagocytic vesicles, and activate autophagy. Beclin1 can regulate the interaction between autophagy and apoptosis and participate in the regulation of autophagy and apoptosis [35]. In our research, we concluded that apelin-13 significantly increased the expression of Beclin1. This indicates that apelin-13 enhances autophagy and decreases apoptosis in vitro models.

The apelin-APJ system is expressed throughout the center of the nervous system and performs a wide range of functions [12]. Several studies on traumatic brain injury and cerebral ischemia-reperfusion injury showed that apelin-13 inhibits apoptosis and plays a neuroprotective role [15,16,17]. In our study, we identified and verified the differential expression of apelin in rat spinal cord injured tissues and normal spinal cord tissues by transcriptome sequencing in vivo and proved that apelin-13 protects neurons by strengthening autophagy and attenuating early-stage postspinal cord injury apoptosis in vitro. Based on these experimental results, we believe that Apelin-13 is expected to become a fresh target for the clinical treatment of spinal cord injury. However, there are still limitations in our study. The in vitro study of apelin-13 is not equivalent to the in vivo study, and therefore it is not yet completely proven that apelin-13 has a protective effect on spinal cord neurons. Future studies may include animal experiments to translationally examine whether apelin-13 has a protective effect on spinal cord neurons in vivo.

## 5. Conclusions

Our study identified and verified the differential expression of apelin in rat spinal cord injured tissues and normal spinal cord tissues by transcriptome sequencing in vivo and proved that apelin-13 strengthens autophagy and debilitates early-stage postspinal cord injury apoptosis in vitro. These results show that apelin-13 may act as a novel therapeutic strategy for SCI.

## Figures and Tables

**Table 1:** Cytotoxicity of Apelin-13 at different concentrations was determined by CCK-8 assay.

Concentration of Apelin-13 (μM)	450 nm OD-Value (Mean ± SD)	Cell Viability Percentage (%) (Mean ± SD)
0	1.113 ± 0.057	100.000
0.001	1.142 ± 0.061	102.900 ± 1.481
0.01	1.173 ± 0.069	105.905 ± 0.719
0.1	1.188 ± 0.080	107.432 ± 0.487
1	1.229 ± 0.098	111.414 ± 2.188
10	1.169 ± 0.042	105.538 ± 2.131

**Table 2:** Determination of cell viability under different conditions by CCK-8 assay.

Group	450 nm OD-Value (Mean ± SD)	Cell Viability Percentage (%) (Mean ± SD)
Control	1.137 ± 0.016	100.000
Control+Apelin-13	1.251 ± 0.010	111.111 ± 4.332
OGD	0.753 ± 0.022	57.680 ± 3.912
OGD+Apelin-13	0.892 ± 0.021	73.451 ± 3.941

## Data Availability

The raw/processed data required to replicate these findings cannot be shared currently as the data also forms part of an ongoing research.
